# Recent Advances in the Targeting of Epigenetic Regulators in B-Cell Non-Hodgkin Lymphoma

**DOI:** 10.3389/fgene.2019.00986

**Published:** 2019-10-16

**Authors:** Marcelo L. Ribeiro, Diana Reyes-Garau, Marc Armengol, Miranda Fernández-Serrano, Gaël Roué

**Affiliations:** ^1^Laboratory of Experimental Hematology, Department of Hematology, Vall d’Hebron Institute of Oncology (VHIO), Vall d’Hebron University Hospital, Autonomous University of Barcelona, Barcelona, Spain; ^2^Laboratory of Immunopharmacology and Molecular Biology, Sao Francisco University Medical School, Braganca Paulista, São Paulo, Brazil

**Keywords:** B-cell lymphoma, DNMT, EZH2, HDAC, PRMT inhibitor, BET bromodomain inhibitor (BETi), combination therapy

## Abstract

In the last 10 years, major advances have been made in the diagnosis and development of selective therapies for several blood cancers, including B-cell non-Hodgkin lymphoma (B-NHL), a heterogeneous group of malignancies arising from the mature B lymphocyte compartment. However, most of these entities remain incurable and current treatments are associated with variable efficacy, several adverse events, and frequent relapses. Thus, new diagnostic paradigms and novel therapeutic options are required to improve the prognosis of patients with B-NHL. With the recent deciphering of the mutational landscapes of B-cell disorders by high-throughput sequencing, it came out that different epigenetic deregulations might drive and/or promote B lymphomagenesis. Consistently, over the last decade, numerous epigenetic drugs (or epidrugs) have emerged in the clinical management of B-NHL patients. In this review, we will present an overview of the most relevant epidrugs tested and/or used so far for the treatment of different subtypes of B-NHL, from first-generation epigenetic therapies like histone acetyl transferases (HDACs) or DNA-methyl transferases (DNMTs) inhibitors to new agents showing selectivity for proteins that are mutated, translocated, and/or overexpressed in these diseases, including EZH2, BET, and PRMT. We will dissect the mechanisms of action of these epigenetic inhibitors, as well as the molecular processes underlying their lack of efficacy in refractory patients. This review will also provide a summary of the latest strategies being employed in preclinical and clinical settings, and will point out the most promising lines of investigation in the field.

## Introduction

### Characteristics of B-Cell Non-Hodgkin Lymphoma (B-NHL)

At the origin of 4% of all cancers and more than 90% of the cases of lymphoma, B-NHLs comprise a heterogeneous group of lymphoid neoplasms. According to the last World Health Organization hematopoietic and lymphoid tumor classification, more than 40 distinct entities are categorized, according to a combination of morphological, immunophenotypic, genetic, and clinical features, having each entity its own clinical course and requiring specific treatments (**Table 1**) (Campo et al., 2011; Scott and Gascoyne, 2014; Swerdlow et al., 2016). Originated from either mature or immature B cells, B-NHLs are characterized by the proliferation of lymphocytes, mainly in lymphoid organs and in extranodal tissues. Their diversity can often be traced to a particular stage of differentiation, from the bone marrow where the normal precursor B cell is originated to secondary lymphoid tissues where B cells undergo multiple rounds of selection before their differentiation into plasma cells or memory B cells. During these processes, the VDJ heavy chain is formed, followed by VJ light-chain gene rearrangement, which allows the pre-B cells to express intracytoplasmic μ-heavy chains. Subsequently, immature immunoglobulin (Ig)-positive B cells are formed. Within the lymph node, and in contact with a determined antigen, naïve B cells can mature into IgM-secreting plasma cells or may proliferate into primary follicles to form germinal center (GC) centroblasts. Upon maturation, they further differentiate into centrocytes, which give place to memory B cells or plasma cells. Within the GC, somatic hypermutation in the *Ig heavy or light chain variable region (IGHV* or *IGHL*) genes leads to increased antigen affinity.

**Table 1:** Classification of B-cell non-Hodgkin lymphoma.

Name	Cell of origin	Genetic aberration	Involved genes	Frequency (%)	References
B-ALL	Hematopoietic stem cell or B-cell progenitor	Hyperdiploidy t(12;21)(p13.2;q22.1) t(1;19)(q23;p13.3) t(9;22)(q34.1;q11.2)	– *ETV6/RUNX1* *TCF3/PBX1* *BCR/ABL1*	31* 28* 9* 5*	(Yeoh et al., 2002)
CLL/SLL	Naïve (unmutated IGHV subset) or memory (mutated IGHV subset) B cell	del(13q14.3) – Trisomy of 12 del(11q)	– *SF3B1* – *ATM*	54 21 14 12	(Haferlach et al., 2007)(Landau et al., 2015)
LPL	Post-follicular B cell	– del(6q) –	*MYD88* – *CXCR4* *ARID1A*	90 43 27 17	(Hunter et al., 2014)
NMZL	Post-germinal center marginal zone B cell	Gains of 3p Gains of 18q del(6q23)	– – *TNFAIP3*	24 24 16	(Rinaldi et al., 2011)
EMZL-MALT	Post-germinal center marginal zone B cell	Trisomy of 3 del(6q23) t(11;18)(q21;q21) Trisomy of 18	– *TNFAIP3* *BIRC3/MALT1* –	31 30 13 11	(Streubel et al., 2004)(Rinaldi et al., 2011)
SMZL	Marginal zone B cell with or without antigen exposure	del(7q) Gains of 3q del(17p13) or mutation –	– – *TP53* *KLF2*	26 20 16 12	(Rinaldi et al., 2011)(Parry et al., 2015)
HCL	Late activated memory B cell	– –	*BRAF* *MAP2K1*	100 48	(Tiacci et al., 2011)(Waterfall et al., 2013)
FL	Germinal center B cell	t(14;18)(q32;q21) – – –	*IGH/BCL2* *KMT2D* *CREBBP* *TNFRSF14*	89 82 64 35	(Horsman et al., 1995)(Okosun et al., 2014)
					
MCL	Peripheral B cell of the inner mantle zone	t(11;14)(q13;q32) Gain of 3q26 del(11q) or mutation del(17p13) or mutation	*IGH/CCND1* – *ATM* *CCND1* *TP53*	57 49 41 34 27	(Beà et al., 1999) (Beà et al., 2013)
DLBCL-GCB	Peripheral mature B cell of germinal center origin	– del(1p36) or mutation Gains of 2p16 t(18q21)	*KMT2D* *TNFRSF14* *REL* *BCL2*	46 38 30 28	(Dubois et al., 2016)(Scholtysik et al., 2015)(Roulland et al., 2018)
DLBCL-ABC	Peripheral mature B cell of germinal center exit or post-germinal center	del(9p21) del(6q21) or mutation – –	*CDKN2A* *PRDM1* *KMT2D* *MYD88*	47 41 41 28	(Scholtysik et al., 2015)(Dubois et al., 2016)
BL	Germinal center B cell	t(8;14)(q24;q32) –Other t(8q24)	*MYC/IGH* *ID3* *TCF3* *MYC*	77 58 29 8–15	(Toujani et al., 2009)(Schmitz et al., 2012)

*Frequency in pediatric cases. B-ALL, B-cell acute lymphocytic leukemia; CLL/SLL, chronic lymphocytic leukemia/small lymphocytic lymphoma; LPL, lymphoplasmacytic lymphoma; NMZL, nodal marginal zone lymphoma; EMZL-MALT, extranodal marginal zone lymphoma of mucosa-associated lymphoid tissue; SMZL, splenic marginal zone lymphoma; HCL, hairy cell leukemia; FL, follicular lymphoma; MCL, mantle cell lymphoma; DLBCL-GCB, diffuse large B-cell lymphoma of germinal center B-cell subtype; DLBCL-ABC, diffuse large B-cell lymphoma of activated B-cell subtype; BL, Burkitt lymphoma.

**Figure 1:**
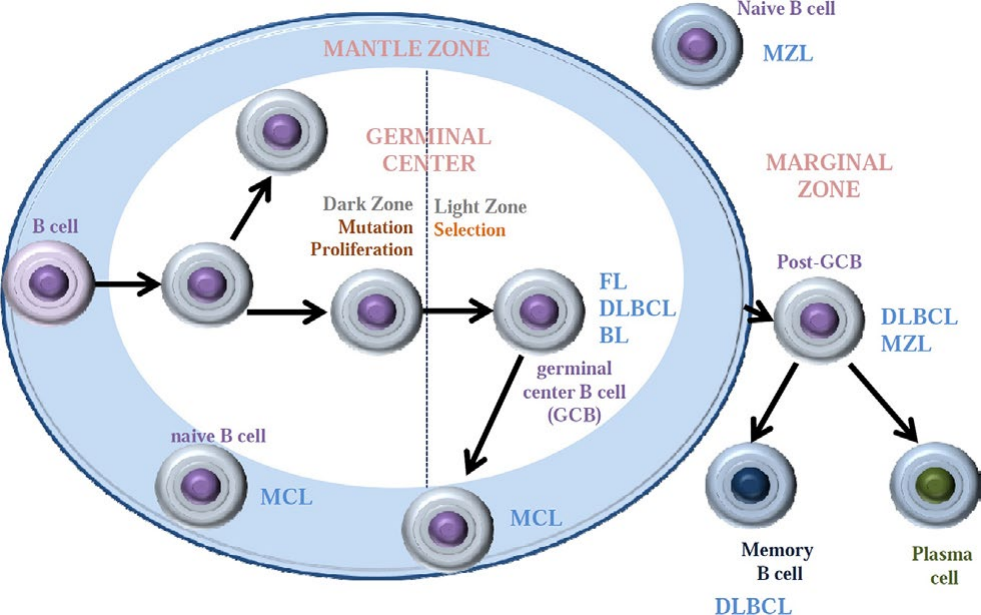
Major B-cell non-Hodgkin lymphoma subtypes arise from different cell of origin within the lymph node. Mantle cell lymphomas (MCL) arise from naive B cells or germinal center (GC) B cells found within the mantle zone. Marginal zone lymphomas initiate from naive B cells or GCB that have entered the marginal zone. GCB are the origin of follicular lymphomas (FL), Burkitt lymphoma (BL), and diffuse large B-cell lymphomas (DLBCL) when still in the germinal center. This last DLBCL appears to also form GCB within the marginal zone or from fully developed memory B cells.

Although tightly regulated, the B-cell differentiation process and especially the antibody diversification phase can be accompanied by inherited events that may favor lymphomagenesis, such as chromosomal translocations, oncogene activation, and/or inactivating mutations in tumor suppressor genes. Infection by determined viruses, such as the Epstein–Barr virus, has also been involved in lymphomagenesis. The malignant counterparts of the early B-cell differentiation steps account for B lymphoblastic lymphomas, which harbor high similarity with B progenitor cells. On the other hand, mantle cell lymphomas (MCLs) and a subset of chronic lymphocytic leukemia (CLL) with unmutated *IGHV* are thought to derive from naive B cells and pre-GC mature B cells expressing the CD5 surface marker. Other GC-originated lymphomas, including follicular lymphoma (FL), Burkitt’s lymphoma (BL), a subset of diffuse large B-cell lymphoma (DLBCL), and Hodgkin’s lymphoma (HL), present mutations in *IGHV* gene. Additional entities, including marginal zone lymphoma (MZL), lymphoplasmacytic lymphoma, CLL with somatic *IGHV* mutation, another subset of DLBCL, and multiple myeloma (MM) correspond to post-GC cells. Each lymphoma subtype retains key features of their cell of origin as judged by the similarity of immunophenotype, histological appearance, and gene expression profiles (Seifert et al., 2013) (**Table 1**). The putative normal B-cell counterpart of each B-cell lymphoma is summarized in **Figure 1**.

In the last decade, loads of evidences have suggested an association between the frequent alterations in chromatin state and epigenetic regulators observed in B-NHL patients, and disease formation and progression.

### Altered Chromatin-Modifying Enzymes in B-NHL

Contrary to the general belief that only accumulations of DNA mutations might lead directly to the development of tumorigenic processes, it has been progressively reported a growing subset of epigenetic alterations lying at the basis of many malignancies, including those occurring in lymph nodes. Interestingly, in B-cell lymphomas, certain somatic mutations in chromatin-modifying enzymes account for several epigenetic alterations, suggesting that an aberrant epigenetic landscape in B-NHL may be a consequence of genetic alterations associated with a particular lymphoma subtype. For instance, deleterious and/or loss of function mutations in the histone acetyltransferase *CREB binding protein (CREBBP)* or the *E1A binding protein 300 (EP330)* have been reported in about 40% of DLBCL and FL patients as well as in other lymphoma subtypes (Morin et al., 2011; Pasqualucci et al., 2011b). Recurrent point mutations in the histone acetyl transferase (HAT) recruiting gene *myocyte enhancer binding factor 2B (MEF2B)* have been also described in 15% of FL and 13% of DLBCL patients with germinal center B cell (DLBCL-GCB) subtype (Morin et al., 2011). Although no mutations have been reported in the genes coding for histone deacetylases (HDACs), several members of this family like *HDAC1*, *2*, and *6* can be overexpressed in DLBCL, in association with a decrease in the DNA accessibility to the transcription machinery (Marquard et al., 2009).

In addition to mutations in chromatin‐regulatory proteins, epigenetic modifications at chromatin level are also commonly observed in B-NHL as a result of profound changes in DNA methylation patterns. Indeed, while hypo- and hyper-DNA methylation status have been linked to the pathogenesis of several cancer subtypes, somatic mutations in epigenetic genes codifying for DNA methylation regulators have been particularly well associated to a repressed chromatin state and to malignant processes in B-NHL (Esteller et al., 2001; Hassler et al., 2013). Among the main reported alterations, activating mutations in *enhancer of zeste homolog 2 (EZH2)*, a histone methyltransferase (HMT) gene, were found in 22% of DLBCL-GCB patients and 7% of FL patients (Morin et al., 2010). Further loss‐of‐function mutations were observed in the *histone-Lysine N-Methyltransferase 2D (MLL2/KMT2D)* gene in about 90% of FL and 30% of DLBCL patients (Morin et al., 2011; Pasqualucci et al., 2011b; Lohr et al., 2012). Concretely, *MLL2* presents a defective SET domain when mutated by either truncation or frameshift mutations, leading to a reduced H3K4 methylation activity (Shilatifard, 2008; Morin et al., 2011; Pasqualucci et al., 2011b; Lohr et al., 2012).

Hence, B-NHL occurrence as a result of disruption in epigenetic mechanisms has generated a strong rationale to target epigenetic and chromatin regulators for drug discovery attempts. To address these alterations, several Food and Drug Administration (FDA)–approved epigenetic-modulating agents, whose clinical use has been mainly restrained so far to other hematological malignancies (Popovic et al., 2013), are now being made available for their evaluation in B-NHL. These agents include the HDAC inhibitors romidepsin (FK228, depsipeptide), vorinostat (suberanilohydroxamic acid, SAHA), panobinostat (LBH589), and belinostat (PXD101); the DNA methyltransferase (DNMT) inhibitors (hypomethylating agents, HMAs) azacitidine (5-azacytidine) and decitabine (5-aza-2′-deoxycytidine); and the isocitrate dehydrogenase (IDH) inhibitors enasidenib (AG-221) and ivosidenib (AG-120) (**Table 2**).

**Table 2:** FDA-approved epigenetic drugs for hematological malignancies.

Agent	Target	Indication	Year of approval	Current development
Azacitidine	DNMT	MDS CMML AML	2004	
Decitabine	DNMT	MDS AML	2006	Atherosclerosis
Vorinostat	HDAC	CTCL	2006	
Romidepsin	HDAC	CTCL PTCL	2009 2011	HIVAutism
Belinostat	HDAC	PTCL	2014	Ovarian cancer CTCL
Panobinostat	HDAC	MM	2015	CML MDS Breast cancer Prostate cancer

DNMT, DNA methyltransferase; HDAC, histone deacetylase; MDS, myelodysplastic syndrome; CMML, chronic myelomonocytic leukemia; AML, acute myeloid leukemia; CTCL, cutaneous T-cell lymphoma; PTCL, peripheral T-cell lymphoma; MM, multiple myeloma.

## Targeting Writer Epigenetic Enzymes

### DNMT Inhibitors

**Figure 2:**
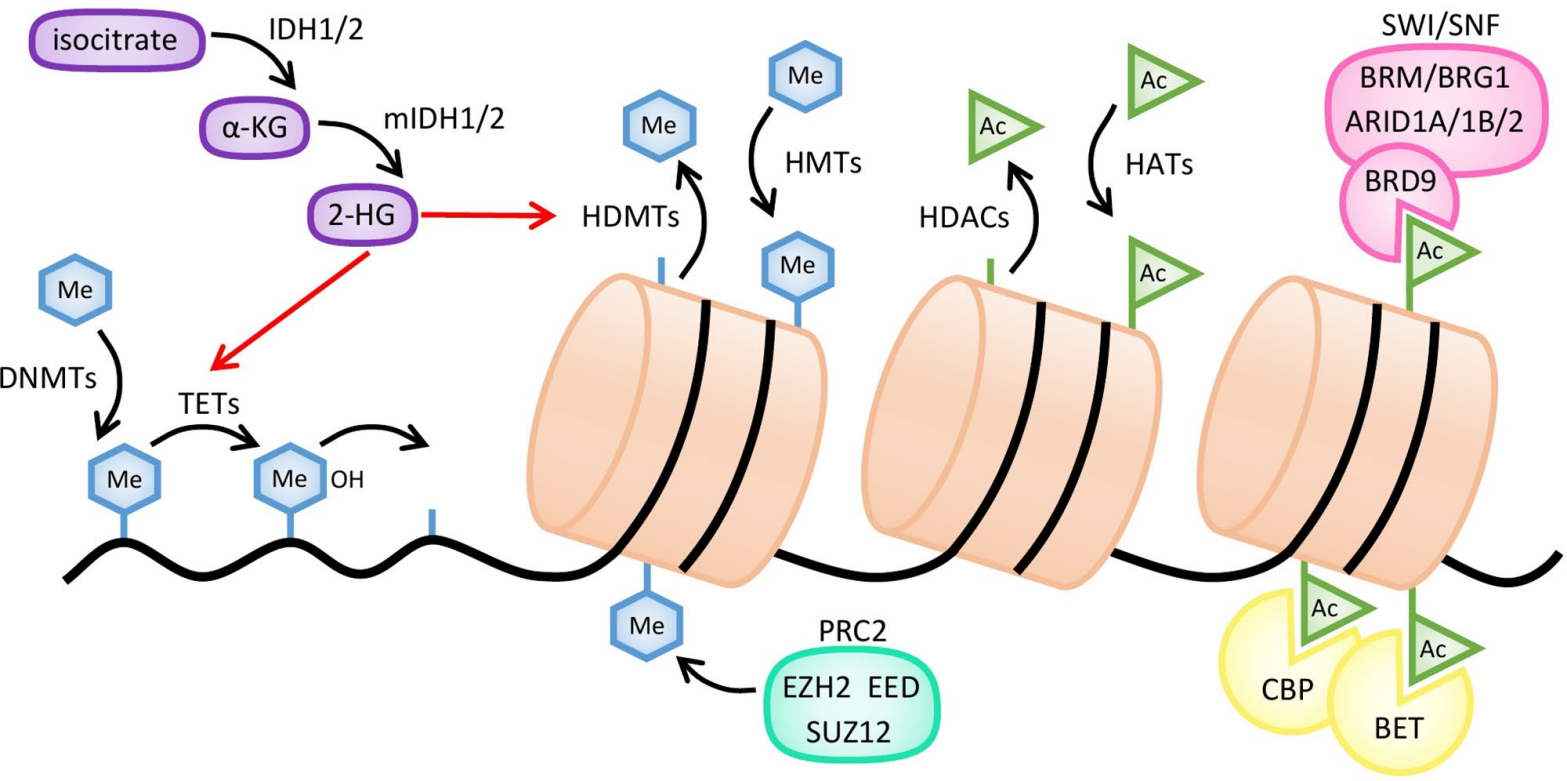
Mechanisms of action of common epigenetic enzymes. Histone methylation is regulated by histone methyltransferases (HMTs), such as the EZH2 subunit of polycomb repressive complex 2 (PRC2), and histone demethylases (HDMTs). DNA methylation is established by DNA methyltransferases (DNMTs) and reversed by several enzymes like TET hydroxymethylases. Demethylation of both histones and DNA is inhibited by 2-hydroxyglutarate (2-HG), produced from α-ketoglutarate (α-KG) by mutant forms of IDH1/2 enzymes (mIDH). Histone acetylation is regulated by histone acetyltransferases (HATs) and histone deacetylases (HDACs). Bromodomain-containing proteins, such as CREB-binding protein (CBP), BET, or the BRD9 subunit of the SWI/SNF complex, bind to acetylated residues of histones.

DNA methylation is responsible for the control of gene expression and for maintaining genomic stability during embryogenesis and tissue differentiation (Meissner, 2010). This process is clonally inherited and preserved in daughter cells, and occurs through the inclusion of a methyl group at cytosine residues in CpG dinucleotides (**Figure 2**). It is carried out by the DNMTs, namely DNMT1 which primarily mediates maintenance methylation during cell division, and DNMT3A and 3B that regulate *de novo* DNA methylation (Belinsky et al., 2003; Hermann et al., 2004). DNA methylation is thought to have a significant role in the regulation of lymphoid compartment, as it has been demonstrated that differential recruitment of DNMT1, DNMT3A, and DNMT3B and consequent specific DNA methylation patterns are determined at early stages during lymphopoiesis and B-cell activation (Shaknovich et al., 2011; Lai et al., 2013).

While on the one hand, DNA methylation is essential for cell homeostasis, on the other hand, disturbance in methylation pattern have been widely described in cancer. Changes in CpG methylation are indeed commonly associated with malignant transformation and tumor progression (Berdasco and Esteller, 2010). In addition, accumulating evidences suggest that aberrant epigenetic regulation, including DNA methylation, exerts an important role in regulating each cancer’s hallmarks (Flavahan et al., 2017). Illustrating this relationship in B-NHL, Shaknovich and collaborators demonstrated the relevance of DNA methylation in defining the molecular DLBCL subtypes (Shaknovich et al., 2010). It was further proposed that DNMT1 and DNMT 3B overexpression may play a role in malignant progression of these tumors (Amara et al., 2010) and also in BL neoplasm (Robaina et al., 2015). In line with this, the disruption of DNA methylation pattern is correlated with disease severity and patient survival in DLBCL and FL (De et al., 2013).

**Figure 3:**
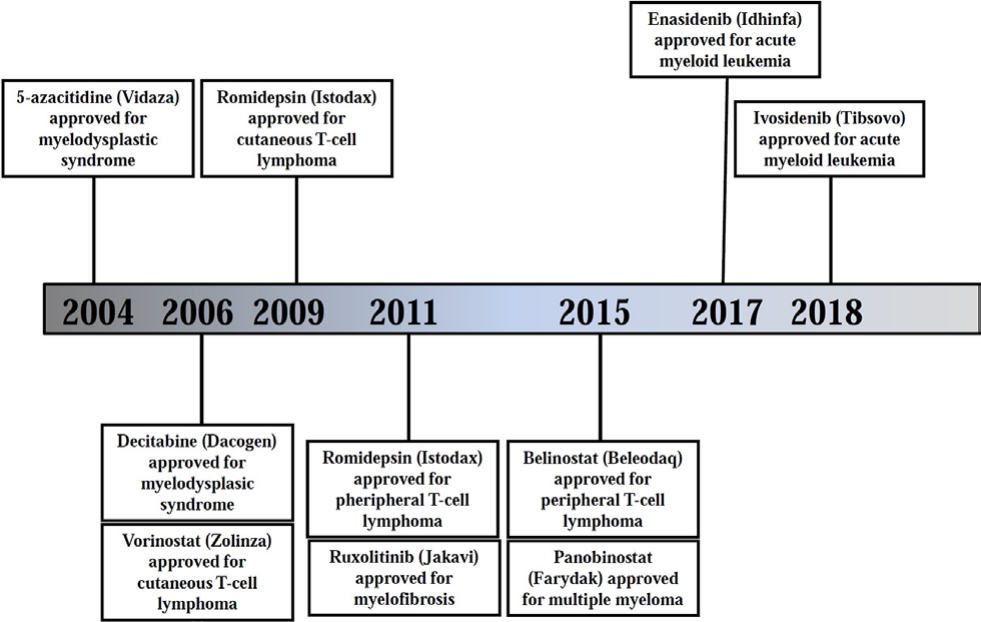
Timeline of FDA approvals of epigenetic-modulating therapies in hematological cancers. Source: https://www.accessdata.fda.gov/scripts/cder/daf/

Considering that the majority of cancers, including B-NHL, harbor an altered DNA methylation pattern, and also taking into account the reversibility of this alteration, the idea to modulate the methylation machinery to restore a “normal” DNA methylation state has attracted great attention in cancer treatment (Azad et al., 2013). The first two DNA methylation epigenetic compounds (DNMTi) ratified by the FDA and the European Medicines Agency for cancer treatment, azacitidine and decitabine (Jones et al., 2016), were initially described as promising chemotherapeutic agents against myelodysplastic syndrome (MDS) and acute myeloid leukemia (AML), although with moderate efficacy and high toxicity (Li et al., 1970; Vogler et al., 1976). In further trials, low-dose decitabine and azacitidine demonstrated to be effective in these patients, improving both the response and the overall survival (OS), leading to their further approval (**Table 2** and **Figure 3**) (Silverman et al., 2002; Fenaux et al., 2009; Lübbert et al., 2016). In B-NHL patients, two phase I studies using decitabine have been completed so far, but the response to therapy and the effect on DNA methylation were moderate (Stewart et al., 2009; Blum et al., 2010). Currently, azacitidine and decitabine are being evaluated alone or in combination in approximately 10 active clinical trials involving relapsed/refractory R/R B-NHL patients (**Table 3**). Considering the preliminary data of these trials, it seems premature to conclude that DNMTis can be used as monotherapy in B-NHL.

**Table 3:** Selected examples of epigenetic drugs under clinical evaluation in B-NHL patients as single agents.

Epigenetic drug class	Drug	Diseases	Sponsor	Trial identifier
HDAC inhibitor	Vorinostat	FL, indolent B-NHL, MCL	Merck Sharp & Dohme Corp.	NCT00875056
	Chidamide (Epidaza)	R/R B-NHL	Sun Yat-sen University	NCT03245905
	Abexinostat	R/R FL	Xynomic Pharmaceuticals, Inc.	NCT03934567
	Mocetinostat (MGCD0103)	DLBCL, FL	Memorial Sloan Kettering Cancer Center and MethylGene Inc.	NCT02282358
	Ricolinostat(ACY-1215)	R/R NHL	Acetylon Pharmaceuticals Inc. and Columbia University	NCT02091063
	Panobinostat	CLL	Peter MacCallum Cancer Centre, Australia	NCT01658241
	CDX101	Advanced lymphomas	Cancer Research UK and Oxford University Hospitals	NCT01977638
IDH1 inhibitor	Ivosidenib(AG-120)	Advanced hematologic malignancies with an IDH1 mutation	Agios Pharmaceuticals, Inc.	NCT02074839
BET bromodomaininhibitor	CPI-0610	Progressive lymphomas	Constellation Pharmaceuticals	NCT01949883
	BMS-986158	Lymphomas	Dana-Farber Cancer Institute	NCT03936465
	Molibresib (GSK525762)	NHL	GlaxoSmithKline	NCT01943851
EZH1/2 inhibitor	DS-3201b	R/R B-NHL	Daiichi Sankyo Co., Ltd.	NCT02732275
	CPI-1205	Progressive B-cell lymphomas	Constellation Pharmaceuticals	NCT02395601
	Tazemetostat	R/R B-NHL	Eisai Co., Ltd.	NCT03009344
	Tazemetostat	R/R NHL with EZH2 gene mutations	National Cancer Institute (NCI)	NCT03213665
PRMT inhibitor	JNJ-64619178	R/R B-NHL	Janssen Research & Development, LLC	NCT03573310
	GSK3326595	MDS, AML	GlaxoSmithKline	NCT03614728
	GSK3368715	DLBCL	GlaxoSmithKline	NCT03666988
DNMT inhibitor	Decitabine	R/R DLBCL	Mingzhi Zhang, Zhengzhou University	NCT03579082
EED inhibitor	MAK683	Advanced DLBCL	Novartis Pharmaceuticals	NCT02900651

EED, embryonic ectoderm development. Source: https://clinicaltrials.gov/.

Although the mechanism of action of DNMTi is not well understood, the activity of decitabine and azacitidine is known to involve their incorporation into the DNA of proliferating cells, followed by irreversible inhibition of DNMT1 enzymatic activity and the addressing of this latest to proteasomal degradation (Ghoshal et al., 2005; Juttermann et al., 2006). Accordingly, two main molecular effects have been described for DNMTi inhibitors: (1) a global demethylation of gene promoters (mainly tumor suppressor genes) and (2) the activation of immune system and the triggering of an anti-tumor immune response (Groudine et al., 1981; Almstedt et al., 2010; Goodyear et al., 2010; Chiappinelli et al., 2015;). As an illustration, in DLBCL it has been described that decitabine can reverse DNA methylation and restore expression of important cancer-related pathways *in vitro* and *in vivo* (Li et al., 2002; Clozel et al., 2013), although in other studies a less drastic and transient effect was observed (Karpf, 2004; McGarvey et al., 2006; Egger et al., 2007). Furthermore, DNMT inhibition is also linked to the demethylation of gene bodies, leading to oncogene downregulation (Wong et al., 2013; Yang et al., 2014).

Several new DNMTis have been developed in the last decade with potential activity in hematological malignancies. Among them, thioguanine (2-amino-1,7-dihydro-6H-purine-6-thione (6-tG)) has been approved by FDA to treat AML patients (Munshi et al., 2014). Its mechanism of action involves its incorporation into DNA, decrease in DNMT activity and DNA methylation, blockade of DNA and RNA synthesis, and ultimately cell death (Hogarth et al., 2008; Yuan et al., 2011; Flesner et al., 2014). Recently described as an experimental DNMTi, 5-fluoro-2′-deoxycytidine (FdCyd) is currently undergoing a phase I/II clinical trial in combination with other drugs (Kinders et al., 2011; Newman et al., 2015). Its mechanism of action involves the ability to block DNMT-dependent DNA methylation (Jones and Taylor, 1980; Beumer et al., 2008). 5,6-Dihydro-5-azacytidine is a reduced, hydrolytically stable form of 5-azacytidine nucleoside (Beisler et al., 1979). The mechanism of action is very similar to that described for azacytidine, with the advantage of a lower toxicity (Avramis et al., 1989). However, its evaluation in clinical settings revealed a reduced response rate and the rise of significant adverse effects (Samuels et al., 1998). Zebularine is another DNMTi, which has been previously described as tumor-selective inhibitor of DNMTs (Cheng et al., 2004). Although there are a lot of evidences, both *in vitro* and *in vivo*, indicating the potential of zebularine as a demethylating agent in a wide range of tumors (Agrawal et al., 2018), its poor bioavailability has prevented its introduction into clinical trials (Ben-Kasus et al., 2005). More recently, 4′-thio-2′-deoxycytidine (TdCyd) and its 5-aza analog, 5-aza-TdCyd, have been reported to downregulate DNMT1 and to exhibit anti-tumor activity *in vitro* and in human leukemia and lung cancer xenografts (Thottassery et al., 2014). Among these last molecules, TdCyd has already entered into phase I clinical evaluation (NCT02423057 and NCT03366116). Further molecules were developed with superior anti-tumoral efficacy and included guadecitabine (SGI-110), a second-generation DNMTi that harbors an improved DNA methylation inhibition in solid tumors both *in vitro* and *in vivo* (Chuang et al., 2010; Srivastava et al., 2015). A phase I clinical trial has provided promising results in patients with MDS and AML (Issa et al., 2015). Fluorocyclopentenylcytosine (RX-3117) is a cytidine analog that presents an anti-tumor activity in a large set of tumor cells and *in vivo*. Its mechanism of action is associated with an inhibition of DNMT1 (Choi et al., 2012). This agent is being evaluated in a phase II study with R/R pancreatic or advanced bladder cancer (NCT02030067).

### EZH2 Inhibitors

EZH2 constitutes the catalytic subunit of the polycomb repressive complex 2 (PRC2). Its structure is composed by a SET domain, typical in chromatin-associated regulators of gene expression (Xiao et al., 2003). It catalyzes histone H3 lysine 27 tri-methylation (H3K27me3) and the subsequent formation of heterochromatic regions and downregulation of the nearby genes (Bracken and Helin, 2009; Ferrari et al., 2014) (**Figure 2**). In B lymphocytes, EZH2 becomes expressed and inhibited in a cyclic manner. First, in pre-B lymphocytes, induction of EZH2 expression is required for an optimal V(D)J recombination. Later on, during the migration to lymphoid tissues, it is downregulated until the GC reaction occurs, after which it becomes re-expressed to allow the silencing of the anti-proliferative genes *cyclin-dependent kinase inhibitor 2A (CDKN2A)* and *cyclin-dependent kinase inhibitor 1A (CDKN1A1)* and the pro-differentiation genes *interferon regulatory factor 4 (IRF4)* and *PR domain zinc finger protein 1 (PRDM1/BLIMP1)* during the somatic hypermutation and isotype switch processes. Finally, EZH2 becomes repressed when mature B cells leave the GC (Velichutina et al., 2010; Béguelin et al., 2013). Gain-of-function mutations in *EZH2* have been reported in several solid tumors and hematological cancers. The consequence of those mutations in GC lymphocytes is the irreversible silencing of certain cell cycle checkpoint and plasma cell differentiation genes (Béguelin et al., 2013). The main gain-of-function mutation identified in DLBCL and FL patients includes a tyrosine deletion (Y641) at the EZH2 SET domain that increases the levels of H3K27me3, promoting a repressed state of cell differentiation and the repression of tumor suppressor genes (Morin et al., 2010; McCabe et al., 2012a). Similar effects have been described as a consequence of the A677G mutation in EZH2, which has been characterized in multiple human lymphoma cell lines. A change in the substrate preferences accounts for the aberrant H3K27me3 levels observed in cells bearing EZH2 mutant forms. Indeed, wt EZH2 displays preference for less methylated substrates whereas Y641 and A667G mutants prefer either substrates with higher methylation levels or show equal affinity for all three substrates (H3K73me0, me1, and me2) (McCabe et al., 2012a). Interestingly, these gain-of-function EZH2 variants expressed in GC B-cell lymphomas seem to synergize with *BCL2* deregulation, favoring the progression of these malignancies (Béguelin et al., 2013). On the other hand, overexpression of wt EZH2 has been also reported in B-NHL (Van Kemenade et al., 2001; Visser and Gunster, 2001), with a positive correlation being observed between *EZH2* transcript levels, tumor aggressiveness, and disease prognosis (Abd Al Kader et al., 2013). Taking into account these considerations, it looks reasonable that inhibiting EZH2 activity could result in a potential therapeutic strategy to treat B-NHL.

In this context, many efforts directed to develop highly selective EZH2 inhibitors have been made in the last decade. EZH2 activity was initially targeted by means of the carbocyclic adenosine analog 3-deazaneplanocin A (DZNep), an inhibitor of *S*-adenosylhomocysteine hydrolase. DZNep promotes a global increase in the levels of 5-adensylhomocystein and a further inhibition in the activity of many methyltransferases, including EZH2. Nevertheless, due to its mechanism of action, it resulted to be too unspecific as many other methyltransferases were similarly affected. In 2012, a small chemical compound named El1 with a good capacity to inhibit the Y641 mutant and wt EZH2 form was evaluated for the treatment of DLBCL. This compound was designed as a competitive inhibitor of the EZH2 methyl group donor *S*-adenosyl--methionine (SAM). Unlike DZNep, El1 showed a 10,000-fold selectivity for EZH2 over other HMTs and a 90-fold selectivity over EZH1 methyltransferase. This compound promoted a global decrease in methyl donor availability, leading to a lower global levels of H3K27me3 (Qi et al., 2012). Other subsequent compounds directed specifically against EZH2 are the dual EZH2/1 inhibitors UNC1999, with a potent capacity to suppress H3K27me3 and H3K27me2 levels and to inhibit proliferation of mixed lineage leukemia (MLL)-rearranged cells, and the OR-S1 and OR-S2 inhibitors, which were assessed for the treatment of DLBCL, AML, and MM (Konze et al., 2013; Honma et al., 2017). Later on, EPZ0005687 and GSK126, two selective and SAM-competitive EZH2 inhibitors with a higher inhibitory capacity for the mutant EZH2 form, were developed and tested in DLBCL and FL (McCabe et al., 2012b; Knutson et al., 2014). In 2014, GSK126 entered into phase I clinical trials with B-NHL and MM patients (NCT02082977) (Zeng et al., 2016; Yap et al., 2018), but unfortunately that study had to be discontinued as a consequence of insufficient therapeutic activity, evidencing the need to keep working in the improvement of those inhibitors. Also in 2014, CPI-360 and its more potent and stable analog, CPI-169, were reported to be effective EZH2 inhibitors for the treatment of several B-NHL subtypes (Vaswani et al., 2016). An improved version of these latest, CPI-1205, showed a higher oral bioavailability and was first tested in preclinical studies with xenograft mouse models generated from human B-NHL cell lines and further challenged in phase I trials for the treatment of DLBCL (NCT02395601).

Valemetostat (DS-3201) is another potent wild-type (wt) and mutant EZH1/2 inhibitor that demonstrated a strong anti-proliferative effect against NHL, DLBCL, and T-cell lymphoma (Maruyama et al., 2017). Currently, tazemetostat (EPZ‐6438), another SAM competitive inhibitor with a high affinity for the wt and the mutant EZH2 forms, is being evaluated in clinical studies to treat R/R B-NHL and MM patients (NCT03456726) (Knutson et al., 2014; Gulati et al., 2018), reaching an overall response rate of 38% in a phase I clinical trial (Italiano et al., 2018).

Despite first promising results, single-agent treatment with EZH2 inhibitors is in general slightly effective in aggressive lymphomas. Among the possible mechanism(s) of resistance, overactivation of the phosphatidylinositol 3-kinase (PI3K) and mitogen-activated protein kinase (MAPK) pathways has been identified in GSK126-resistant DLBCL cells (Bisserier and Wajapeyee, 2018). Thus, it looks reasonable to prioritize the discovery of new drug combination associating EZH2 inhibitors with other compounds targeting key signaling pathways in order to prevent and/or overcome the occurrence of EZH2i resistance in lymphoid neoplasm with mutated EZH2.

### PRMT Inhibitors

A conserved biological mechanism within all eukaryotic organisms, from yeast to higher mammals, is arginine methylation (Migliori et al., 2010). This post-translational modification is mediated by *N*-arginine methyltransferases (PRMTs), which catalyze the transfer of a methyl group, from SAM to the omega nitrogens found in terminus guanidine group of an arginine residue of the side chain. This transfer may occur in one or both nitrogens (Bedford and Clarke, 2009). Among the nine different members of the PRMT family (Schubert et al., 2003), PRMT1 is the major enzyme responsible for arginine methylation followed by PRMT5, according to the observation that PRMT1 and PRMT5 knockout mice die at an early stage during development whereas mice lacking any of the other seven PRMTs are fully viable (Hadjikyriacou et al., 2015). Protein modifications performed by PRMTs are traditionally related to important genetic processes such as DNA repair and gene transcription, among others. More recently, PRMT functions have been linked to carcinogenesis and metastasis, giving to these enzymes the status of potent therapeutic targets in a variety of cancers where they are overexpressed, including colon, breast, prostate, and lung cancers, neuroblastomas, leukemias, and B-cell lymphoma (Yoshimatsu et al., 2011).

Within this family, upregulation of PMRT1 and PRMT5 has been widely associated with hematological malignancies (Greenblatt et al., 2016; Smith et al., 2018). In particular, the expression and function of PMRT5 have been extensively examined during lymphomagenesis, as this enzyme is highly expressed in primary samples and cell lines from different leukemia and lymphoma subtypes, where it promotes the repression of tumor suppressors such as the retinoblastoma proteins. In these models, experimental studies have suggested that PRMT5 upregulation may be caused by overexpression of *MYC* and *NOTCH* oncogenes (Wang et al., 2008). In transformed DLBCL, the *S-methyl-5’-thioadenosine phosphorylase (MTAP)* gene encoding for a critical methionine metabolism enzyme is deleted due to its proximity to the tumor suppressor gene *CDKN2A* (Dreyling et al., 1998), and this phenomenon sensitizes cancer cells to PRMT5 inactivation (Marjon et al., 2016). A remarkable interplay has also been described between PRMT5 and the *B cell lymphoma 6 (BCL6)* oncogene during the lymphomagenesis in the GC (Lu et al., 2018), suggesting that pharmacological inhibition of arginine methylation could be of special interest in BCL6-driven lymphoma. Regarding PRMT1, an interesting interaction exists between this enzyme and EZH2 in DLBCL-GCB tumors. Indeed, recent works have reported an increase in PRMT1-related histone arginine methylation in DLBCL-GCB cells resistant to EZH2 inhibition, in association with BCL-2 overexpression and modulation of the B-cell receptor (BCR) downstream signaling, supporting the rational association of EZH2 and PRMT1 inhibitors in DLBCL patient samples (Goverdhan et al., 2017).

Among the multiple functional inhibitors that have been developed to target the different members of the family, PRMT1 and PRMT5 small molecule inhibitors have already shown great potential against B-NHL, either alone or upon their combination with other agents. As an illustration, promising results have been obtained with the specific PRMT5 inhibitor EPZ015666 (GSK3235025) when used as single agent in *in vitro* and *in vivo* models of MCL (Chan-Penebre et al., 2015).

## Targeting Eraser Epigenetic Enzymes: HDAC Inhibitors

By favoring an open chromatin state, histone acetylation allows numerous transcription factors to bind DNA and to activate gene expression. At the same time, acetylated histones increase DNA accessibility to transcriptional activators and counteract the function of transcriptional repressors (McClure et al., 2018). Acetylation of histones and non-histone proteins is regulated through a correct balance between HAT and HDAC activities. Among these enzymes, the most advanced subfamily is human HDACs, which have been classified into four classes according to their sequence homology, activity, and subcellular localization. HDACs 1, 2, 3, and 8 constitute class I. HDAC 4, 5, 6, 7, 9, and 10 belong to class II. Class III includes sirtuin 1 (SIRT1) and sirtuin 7 (SIRT7), two NAD-dependent structurally unrelated protein deacetylases (Minucci and Pelicci, 2006). Finally, class IV is represented by HDAC11. In contrast to class II HDACs which show a heterogeneous expression pattern, class I HDACs are found at particularly high levels in lymphoid cell lines and primary tumors, suggesting a predominant role of these latest in lymphomagenesis. Accordingly, the design of HDAC inhibitors (HDACis) in lymphoid malignancies has been mainly centered on this latest group of enzymes (Gloghini et al., 2009).

Several structurally distinct classes of HDACis have been developed. These molecules can be divided into five chemical groups: hydroxamic acids, cyclic peptides, electrophilic ketones, short-chain fatty acids, and benzamides. Pan-HDACis have the capacity to inhibit almost all HDACs with the exception of class III HDACs and include the hydroxamic acid derivatives vorinostat, givinostat (ITF2357), abexinostat, panobinostat, belinostat, and trichostatin A, the carboxylate sodium butyrate, and the cyclic peptide trapoxin (Bradner et al., 2010; Di Costanzo et al., 2014). Taking into account that HDACs can also modulate the function of several non-histone proteins regulating a number of physiological processes (Lane and Chabner, 2009), and that HDACs can simultaneously exert pro- and anti-leukemic activities (Heideman et al., 2013; Santoro et al., 2013), blocking individual HDACs with isotype-selective inhibitors specific for one or two classes of HDACs might represent a strategy of choice for the treatment of lymphoid tumors. In line with this, the isotype-selective HDACis include the benzamides entinostat (MS-275, SNDX-275) and mocetinostat (MGCD0103) (Fournel et al., 2008; Vannini et al., 2004), the hydroxamic acid derivative rocilinostat (ACY-1215) (Santo et al., 2012), and the cyclic peptide romidepsin, which show preference for HDAC1-6-8, HDAC6, and HDAC1-2, respectively (Lemoine and Younes, 2010). Several HDACis like vorinostat, mocetinostat, and entinostat can be administered orally; conversely, other agents like romidepsin are given intravenously (Batlevi et al., 2016; Mann et al., 2007; Younes et al., 2011; Holkova et al., 2017). By inhibiting the catalytic activity of their target HDAC(s), these compounds impair the formation of HDAC–substrate complexes, thus altering the transcriptomic pattern of the malignant cells as well as the activity of non‐histone proteins, ultimately leading to growth arrest, differentiation, and induction of apoptosis (Qiu et al., 2000). Of importance, when compared to their malignant counterparts, healthy tissues are generally unaffected by HDACis (Mai et al., 2005).

A number of preclinical studies have highlighted a role for HDACi therapy in a range of B-cell lymphoma, including DLBCL, HL, and BL, either alone or in combination with other epidrugs such as HMAs, with small molecule agents or with standard chemotherapeutics (Buglio et al., 2008; Kretzner et al., 2011; Kewitz et al., 2012; Ageberg et al., 2013; Klein et al., 2013; Rozati et al., 2016; Garrido Castro et al., 2018). Among these studies, the weak HDACi valproic acid was shown to overcome DLBCL cell resistance to the standard R-CHOP (rituximab, cyclophosphamide, doxorubicin, vincristine, prednisone) chemotherapeutic regimen (Ageberg et al., 2013). In preclinical models of DLBCL and MCL, panobinostat, belinostat, depsipeptide, and vorinostat were shown to evoke tumor growth arrest, differentiation, and/or apoptosis *in vitro* and/or *in vivo*, mediated by the accumulation of DNA damage upon PARP trapping (Valdez et al., 2018), G1 cell cycle arrest consequent to an increase in the expression of the cyclin-dependent kinase inhibitor p21, acetylation of histone H3 (Xue et al., 2016), or transcriptional activation of the BCL-2 family proapoptotic members BIM, BMF, and NOXA (Kalac et al., 2011; Xargay-Torrent et al., 2011).

Based on these preclinical studies, several HDACis have entered clinical trials under different modalities (monotherapies or in combination). Many of these trials have been conducted in DLBCLs, FLs, and HLs using HDACis, either alone or in combinatorial therapies (Watanabe et al., 2010; Stathis et al., 2011; Younes et al., 2012; Oki et al., 2013; Ogura et al., 2014; Chen et al., 2015; Morschhauser et al., 2015) (**Table 2** and **Figure 3**). As monotherapy, HDACis have shown a wide range of response in lymphoma patients, varying from complete remissions (CRs) to no response. In the absence of biomarkers for prediction of clinical outcome, the molecular mechanisms of resistance are poorly understood. Vorinostat was the first proved in relapsed B-NHL patients, including FL, MZL, and MCL. In a phase II study including relapsed FL, non-FL indolent NHL and MCL patients, oral vorinostat showed low levels as a single agent, with the exception of FL, in which an overall response rate (ORR) of 47–49% (referring to the proportion of patients with tumor size reduction of a predefined amount and for a minimum time period) and a CR rate of 23% was observed (Kirschbaum et al., 2011; Ogura et al., 2014). This agent was also tolerated, but displayed limited activity in another phase II trial against R/R DLBCL, with only 1/18 patients presenting complete response (Crump et al., 2008).

With the pan-HDACis abexinostat and quisinostat, or the class-specific mocetinostat and entinostat, the response rates were quite variable (from 12% to 56%), and mostly dependent on the drug and on the lymphoma subtype. The most robust responses were obtained with abexinostat in FL patients (56% ORR). This latest drug showed a unique pharmacokinetic profile and an optimized oral dosing schedule that allowed for a superior anti-tumoral activity. In a recent phase II study with patients with R/R B-NHL or CLL, among the evaluable patients the ORR was 28%, with highest responses observed in FL patients (ORR 56%) and DLBCL (ORR 31%) (Ribrag et al., 2017). A phase II clinical trial with mocetinostat in patients with R/R DLBCL and FL showed promising results (Batlevi et al., 2017), whereas for entinostat only one B-NHL patient has been included in phase II trial; therefore, no conclusion can be made on its efficacy in this subgroup of patients (Kummar et al., 2007).

Similar to DNMTis, the effectiveness of the first-generation HDACis carries significant toxicity and is limited to hematopoietic malignancies, which makes them challenging to combine (Suraweera et al., 2018). It is believed that part of this toxicity may be related to the capacity of HDACis to alter directly the function of many non-histone proteins. Toxicity may also be due to widespread activity across HDAC isoforms; therefore, the focus of second-generation HDACi discovery was to enhance the discrimination over HDAC family members (Galli et al., 2010; Knipstein and Gore, 2011; Younes et al., 2011; Santo et al., 2012; Evens et al., 2016). In this context, targeting HDAC6 was associated to the upregulation of CD20 and consequent enhanced efficacy of anti-CD20 monoclonal antibody therapy (Bobrowicz et al., 2017). Also, tucidinostat (CS055/chidamide), the first oral subtype-selective HDACi, was approved for the treatment of refractory/relapsed PTCL by the China Food and Drug Administration. This compound inhibits HDAC1, HDAC2, HDAC3, and HDAC10, and has entered a phase II clinical trial as single-agent treatment for patients with R/R B-NHL (NCT03245905) based on preliminary evidences of clinical activity in DLBCL (Yang et al., 2018).

Another approach to maximize efficacy with manageable toxicity consists in developing dual inhibitors. In this field, CUDC-907, a novel first-in-class oral small molecule inhibitor of both HDAC (class I and II) and PI3K (class Iα, β, and δ), has demonstrated excellent levels of activity (55% ORR) and tolerability in DLBCL patients in a phase IA clinical trial (Younes et al., 2016). In a second phase IB trial, the drug has been tested in patients with R/R DLBCL and showed a response rate of 37%, with a higher effect in MYC-altered *versus* MYC-unaltered patients (Oki et al., 2017). As a result of these encouraging initial data, this agent is currently being evaluated in a phase II study including DLBCL patients, and also in a phase I trial involving pediatric patients with lymphomas (NCT02674750 and NCT02909777).

## Targeting Reader Epigenetic Enzymes

### BET Inhibitors

Among the post-translational modifiers with ability to orchestrate chromatin organization, bromodomain (BD)-containing proteins are readers of Ac-K residues at the N-terminal histone tails. They act as scaffolds that enable histone attachment to the chromatin and form active multi-protein transcription complexes, thereby modulating chromatin dynamics and ultimately diversifying gene expression (Filippakopoulos et al., 2012; Chaidos et al., 2015; Smith and Zhou, 2016). This family of proteins contains 46 members, comprising nuclear proteins with HAT or HMT activity, chromatin remodelers, helicases, transcription co-activators, and mediators or scaffold proteins. They are subdivided into eight subfamilies (I to VIII), based on their structure and sequence similarities. Subfamily II is the most studied one and includes the bromodomain-containing proteins mBRDT, BRD2, BRD3, and BRD4 (Padmanabhan et al., 2016). Besides the presence of two bromodomains (BD1 and BD2) that allow acetylated chromatin recognition, these proteins harbor an extra-terminal domain, which is responsible for protein–protein interactions. This bromodomain and extra-terminal (BET) subfamily has thus the capacity to act as protein adaptors facilitating the recruitment of chromatin remodelers and transcription factors for further initiation and elongation of transcription (Delmore et al., 2011; Chaidos et al., 2015; Padmanabhan et al., 2016). Several reports have highlighted the importance of the BET proteins action over DNA enhancers for the regulation of certain oncogenes expression (Lovén et al., 2013). Altogether, these studies make BET proteins attractive therapeutic targets in cancer.

As interfering with this family of proteins may serve as a strategy to address transcription irrespective of the presence of epigenetic mutations, BET proteins inhibitors have been a significant area of focus in the last decade, in cancer but also in inflammation, fibrosis, and heart diseases (Vakoc, 2015). Drug developmental studies have paid special attention to the Ac-K binding sites in the bromodomains, as these deep hydrophobic pockets with conserved asparagine and/or aspartate residues make BET proteins highly druggable (Cox et al., 2016). Indeed, the most common drug targeting approach in this family has been the development of small molecules that could block the lysine-binding pocket and disrupt the interactions between BDs and the Ac-K on chromatins (Smith and Zhou, 2016).

In 2005, a first bromodomain inhibitor developed by the Zhou laboratory, namely NP1, has the ability to target the BD of the P300/CBP-associated factor transcriptional coactivator (Zeng et al., 2005). This step was followed by the discovery in 2006 of MS7972, a weakly binding fragment specific for CREBBP-BD, hindering its binding to acetylated p53 (Sachchidanand et al., 2006). Among BET proteins, the first target considered to be druggable was BRD4, as a pioneering RNAi base unveiled its critical role in the maintenance of AML. In this study, authors found that BRD4-dependent transcriptional activity could be efficiently targeted by the pan-BET thieno-triazolo-1,4-diazepine (+)-JQ1 (Filippakopoulos et al., 2010; Zuber et al., 2011). This class of diazepine-based small molecule inhibitors, which also includes the benzodiazepine I-BET151 (GSK1210151A) (Dawson et al., 2011) and I-BET762 (GSK525762) (Mirguet et al., 2013) (NCT01943851), utilizes the methyltriazolo-diazepine ring system as the acetyl-mimetic. Further studies demonstrated that inhibition of BRD4 by (+)-JQ1 unveiled the MYC downregulation and, consequently, a genome-wide inhibition of its target genes (Filippakopoulos et al., 2010; Delmore et al., 2011). These results underlined significant preclinical activity of this inhibitor in MYC-driven B-NHL, including the aggressive, so-called “double hit” lymphoma (DHL), characterized by simultaneous oncogenic activation of *MYC* and/or *BCL2/BCL6* (Johnson-Farley et al., 2015). Accordingly, (+)-JQ1 could increase survival of mice xenografted with MYC-driven lymphoma, including those ones bearing either TP53 deletions or intrinsic resistant to the topoisomerase II inhibitor etoposide (Hogg et al., 2016).

These promising results from (+)-JQ1 encouraged the development of BET inhibitors with similar chemical structure, including the BRD4 inhibitor CPI203 characterized by a higher bioavailability profile in mice (Normant et al., 2012; King et al., 2013). This agent displayed remarkable efficacy in different preclinical models of B-NHL, either as single agent or in combination with the BCL-2 antagonist venetoclax in DHLs (Esteve-Arenys et al., 2018), in DLBCL-ABC (Ceribelli et al., 2014) and in both ABC and GCB subtypes of DLBCL in combination with blockade of the CXCR4 chemokine receptor (Recasens-zorzo et al., 2018). In these studies, BRD4i activity was mainly related to the blockade of MYC transcriptional program. This is of special interest, as despite its central role in multiple hematological malignancies, including various subtypes of B-NHL, direct targeting of MYC was considered impossible until the demonstration that BET inhibition could regulate MYC activity in varied contexts, thanks to alleviation of BRD4 occupancy on MYC super-enhancers. Importantly, beside MYC, different anti-apoptotic proteins like BCL-2 and MCL-1 are also downregulated, either by direct transcription repression or as a downstream consequence of BRD4 antagonism (Vakoc, 2015). Unlike the expected general effects of BET inhibition in the elongation of transcription of several genes, changes in the expression of only a small subset of genes was observed in cultures and/or animals receiving this therapy, suggesting that bromodomain inhibitors might be suitable modulators of certain disease-associated genes. As an illustration, high levels of BRD4 co-localize in CLL cells with super-enhancer sites of genes and microRNAs belonging to the BCR-mediated signaling pathway with possible tumor-initiating activity, including *miR-21*, *miR-15*, *TCL1*, *IL21R*, and *IL4R*. Accordingly, in a mouse model of CLL, exposure to the BET inhibitor PLX51107 promoted an expression downmodulation of several tumor-associated genes, followed by consistent reduction in tumor burden (Ozer et al., 2018).

According to these promising results, in the last years a number of clinical leads have entered into trials for the treatment of hematological patients. Nevertheless, several side effects have been reported including some bone marrow and gastrointestinal toxicity that has forced to dose discontinuation or reduction. Nowadays, 18 BET inhibitors are being assessed in clinical trials either as single agents or in combination with other compounds (**Table 4**). While the data from various solid tumor trials look mitigated, several BETis including birabresib (OTX015, MK-8628), molibresib (GSK525762), RO6870810/TEN-010, and mivebresib (ABBV-075) have demonstrated remarkable clinical efficacy in myeloproliferative disorders, while other small molecule inhibitors such as PFI-1, BI-894999, FT-1101, INCB-54329, and CPI0610, a pharmacological derivative of CPI203, are currently undergoing human clinical trials in these patients (**Table 3**). Among these different molecules, molibresib has demonstrated an 18.5% ORR in various subtypes of NHLs including a CR in a DLBCL case (Dickinson et al., 2018). CPI0610 has also been evaluated in a phase I clinical trial (NCT01949883) in 64 R/R FL, DLBCL, or HL patients, showing leading to a complete remission in one FL case and in four DLBCL patients (Blum et al., 2018). In addition, the compound INCB057643 is currently being tested in a third phase I trial involving lymphoma patients, including some FL and DLBCL cases. In this evaluation trial, a CR has been achieved in one FL case whereas in two other patients, the disease has been stabilized (Forero-Torres et al., 2017). In the dose-escalation, open-label, phase I study with OTX015, a 47% complete remission was reported in 17 DLBCL cases; however, objective responses were seen in only three DLBCL patients and clinical activity in other six B-NHL patients (NCT01713582) (Amorim et al., 2016). More recently, the BETis molibresib, CC-90010, and INCB054329 are being challenged in clinical trials including various hematological malignancies (NCT02431260, NCT01943851, and NCT03220347), but no data have been released so far.

**Table 4:** Drug combinations with non-approved epigenetic agents in B-NHL.

Epigenetic drug class	Drug	Agent used in combination	Trial identifier
HDAC inhibitor	CUDC-907	Rituximab, venetoclax, and bendamustine	NCT01742988
	Entinostat	Isotretinoin	NCT00098891
		Molibresib	NCT03925428
	Mocetinostat	Azacitidine	NCT00543582
EZH2 inhibitor	Tazemetostat	Fluconazole, omeprazole, and repaglinide	NCT03028103
		Atezolizumab and obinutuzumab	NCT02220842
		Prednisolone	NCT01897571
	PF 06821497	SOC	NCT03460977
BET inhibitor	Molibresib	Entinostat	NCT03925428
	RO6870810	Venetoclax and rituximab	NCT03255096
INCB057643	Gemcitabine, paclitaxel, rucaparib, abiraterone, ruxolitinib, and azacitidine	NCT02711137	
	FT-1101	Azacitidine	NCT02543879

Source: https://clinicaltrials.gov/.

Although at the moment most of the tested compounds aimed at inhibiting BET bromodomains are pan-BET inhibitors, many efforts are being focused in targeting BET proteins in a more specific and novel way. These new approaches include ABBV-744 (which targets bromodomain-containing protein II) (Sheppard et al., 2018), the bivalent BET inhibitors AZD5153 and MT1 (a JQ1-derived BETi) (Rhyasen et al., 2016; Tanaka et al., 2016), and the so-called BET-PROTACs (QCA570, dBET6, BETd-260, and ARV-771) that drive BET proteins to their degradation by proteolysis-targeted chimera (Raina et al., 2016; Winter et al., 2017; Qin et al., 2018 ). These molecules have shown both to promote apoptosis in MCL-derived cells resistant to the first-in-class Bruton’s kinase (BTK) inhibitor ibrutinib as well as to increase survival compared to OTX015-treated MCL xenografts (Sun et al., 2018). Although promising results have been reported for this new generation of BET-targeting agents in preclinical studies, their therapeutic window when moving to clinical trials has still to be evaluated.

### Non-BET Bromodomain-Containing Proteins: the Histone Acetyltransferase CREB-Binding Protein (CBP)

As previously mentioned, chromatin modifications can regulate several important features of cell function. Among these modifications, histone lysine acetylation is generally associated with activation of gene expression (Shahbazian and Grunstein, 2007). HAT enzymes can deposit acetyl marks on histones and modify chromatin structure. Such marks are also recognized by bromodomains, thus adding a second level of regulation of the transcription process (Kouzarides, 2007). The transcriptional co-activators CBP/p300 are highly homologous, multifunctional proteins that encode a single bromodomain each and possess HAT activity (Chen and Li, 2011; Delvecchio et al., 2013). CBP/p300 act as transcriptional co-factors, involved in the regulation of several biological processes (Dancy and Cole, 2015). Animal studies have shown that CBP and p300 are required for the generation and activity of normal hematopoietic stem cells as well as for adult hematopoietic stem cell maintenance and function (Chan et al., 2011; Rebel et al., 2002). Consequently, CBP ablation has a direct impact on the quiescence, apoptosis, and self-renewal of adult hematopoietic stem cells (Chan et al., 2011) and CBP/p300 have a tumor suppressor role in mice models (Kung et al., 2000; Kang-Decker et al., 2004; Chan et al., 2011). This role of CBP and p300 as tumor suppressors has been also observed in B-NHL, where its inactivating mutation is a common event in FL and DLBCL, providing a rationale for employing drugs with the capacity to modulate acetylation and deacetylation processes in these tumors (Cerchietti et al., 2010; Mullighan et al., 2011; Pasqualucci et al., 2011a).

## Chromatin Remodelers: SWI/SNF and BRG1 and ARID1

The SWItch/Sucrose Non-Fermentable (SWI/SNF) complex was initially discovered in yeast. It is composed by polypeptides associated with a subset of proteins codified by the SWI1, SWI2, SNF2, SWI3, SWI5, and SWI6 genes (Pazin and Kadonaga, 1997). This complex regulates gene transcription by altering DNA–nucleosome interactions at expenses of ATP consumption, thus facilitating or impeding the accession of the transcription machinery at concrete genomic regions (Workman and Kingston, 2002). Several studies have reported its capacity to repair nucleotide excisions and DNA double-strand breaks by homologous recombination (Chai et al., 2005). The mammalian analog of the SWI/SNF complex (mSWI/SNF) is the BRG1-Associated Factors (BAF) complex. It comprised approximately 11 subunits encoded by 19 distinct genes assembled in different combinations according to its specific molecular mechanism of action, and in a concrete genomic region. Two of the BAF components are the human Brahma (hBRM, also SMARCA2) and the Brahma-related gene 1 (BRG1, also SMARCA4). These proteins are ATPase subunits (Khavari et al., 1993) and either one or the other constitute the core component of the BAF complex. They contain BDs within their structure that recognize and contact acetyl groups present in histone proteins (Wang et al., 1996). Although they share similarities in their domain composition, they interact with different families of transcription factors what confers to them specific functions in the BAF complex (Kadam and Emerson, 2003).

BRG1 has been reported to be the most frequently mutated protein of the BAF complex in cancer. Classically, it has been described as a tumor suppressor gene as inactivating mutations of its protein have been found in numerous solid tumors like breast, lung, gastric, bladder, colon, ovarian cancers, and melanomas (Atlas et al., 2012; Hodis et al., 2012; Jelinic et al., 2014), but also in determined B-NHL subtype like DLBCL and MCL (Cuadros et al., 2017). Concretely, these loss-of-function mutations lead to the upregulation of the pro-survival gene *BCL2L1* in MCL, conferring to this malignancy primary resistance to treatment or eventually relapse after dual exposure to ibutrinib and venetoclax (Agarwal et al., 2019). Other studies described BRG1 as a potent oncogene, since its function was required for AML progression in mice, through its binding to *MYC* enhancer region and consequent aberrant expression of this second oncogene (Shi et al., 2013; Buscarlet et al., 2014).

Beside BRG1, several BRG-/BRM-associated factors (BAF subunits) participate in tumoral progression. Two of these subunits, namely the AT-Rich Interaction Domain 1A (ARID1A/BAF250A) and its homologous ARID1B/BAF250B, contain domains capable of recognizing and binding to AT-enriched genomic regions and C terminus region, stimulating the activation of transcription in a glucocorticoid receptor-dependent manner. The presence of each of them in the complex is mutually exclusive, suggesting specific roles at concrete genomic regions (Wang et al., 2004).

Mutations that truncate the ARID1A sequence and promote its degradation have been widely characterized in endometrial carcinoma (Kandoth et al., 2013), colon cancer (Atlas et al., 2012), stomach cancer (Wang et al., 2011), bladder cancer (Gui et al., 2011), neuroblastoma (Sausen et al., 2013), and pancreatic or hepatocellular carcinoma (Biankin et al., 2012; Fujimoto et al., 2012), evidencing the role of this protein in preventing tumoral progression. Similar to the mutations reported for ARID1A, *truncating mutations have also been identified for ARID1B* although in a lesser frequency and *most of them associated with neurodevelopmental disorders* (Santen et al., 2012) *or neuroblastomas* (Lee et al., 2017). ARID1B knockdown has been reported to destabilize the SWI/SNF complex and inhibit cell proliferation in both ARID1A-mutant cancer cell lines and primary tumor cells, suggesting that this protein could constitute an interesting therapeutic target for the treatment of ARID1A-mutant tumors (Helming et al., 2014).

## Indirect Inhibition of Epigenetic Dysregulation by IDH Inhibitors

The enzyme isocitrate dehydrogenase (IDH) catalyzes the conversion of isocitrate into α-ketoglutarate (α-KG) by oxidative decarboxylation using NADP^+^ as a cofactor. The IDH1 isomer is located in the cytosol and the peroxisomes, whereas IDH2 is found in the mitochondria. IDH enzymes play an important role in the tricarboxylic (TCA) or Krebs’ cycle, but are also related with other cellular functions such as the regulation of redox balance (Dang et al., 2016; Dang and Su, 2017). Mutations in *IDH* genes are most commonly found in the R132 codon of *IDH1* and the R172 and R140 codons of *IDH2*, which correspond to evolutionarily conserved residues in the enzyme active site which is critical for substrate binding. Mutant forms of IDH have much lower catalytic activity and are associated with metabolic alterations. More importantly, mutant IDH enzymes gain neomorphic activity as they convert α-KG into 2-hydroxyglutarate (2-HG). Under homeostatic conditions, 2-HG is only produced by errors in catalysis and it is maintained at low levels due to the action of 2-HG-hydroxigenases (2-HGHD). Unlike in bacteria and plants, 2-HG has no known physiological function in mammals (Dang and Su, 2017). 2-HG is structurally similar to α-KG and acts as a competitive inhibitor, blocking the activity of α-KG-dependent dioxygenases. This group of enzymes includes the TET family of hydroxylases, which participate in DNA demethylation, and the JMJ domain-containing histone demethylases (Dang and Su, 2017). The consequent aberrant hypermethylation of both DNA and histones has been associated to a blockade in differentiation in hematopoietic cells (Figueroa et al., 2010; Losman et al., 2013), hepatocytes (Saha et al., 2014), and mesenchymal stem cells (Jin et al., 2015), among other cell types.

Homozygous missense mutations in both *IDH1* or *IDH2* have been described in several cancer types, including glioma, cholangiocarcinoma, and hematological tumors, such as AML and MDS (Dang et al., 2016). Although infrequent, mutations have also been found in lymphoid malignancies like angioimmunoblastic T-cell lymphomas (Cairns et al., 2012) and acute lymphocytic leukemia, both in pediatric (Andersson et al., 2011; Tang et al., 2012) and adult cases (Kang et al., 2009; Abbas et al., 2010; Zhang et al., 2012). Dysregulation of the IDH pathway has also been reported in CLL, as leukemic B cells from these patients show overexpression of IDH1 and lower levels of IDH2 when compared to healthy B cells (Van Damme et al., 2016).

Two IDH inhibitors have been recently approved by the FDA for the treatment of R/R AML in adults. Enasidenib (AG-221) targets IDH2 with R172S, R172K, and R140Q mutations, whereas ivosidenib (AG-120) targets IDH1 with susceptible mutations, such as R132H and R132C (Han et al., 2019). Other non-approved IDH inhibitors are currently in clinical trials involving patients with advanced hematological cancers. Among these molecules, AG-881 is a pan-inhibitor of both IDH1 and IDH2 that can penetrate the blood–brain barrier, while IDH305, FT-2102, and BAY-1436032 are IDH1-specific inhibitors (Dang et al., 2016; Montalban-Bravo and DiNardo, 2018). At the preclinical level, the pharmacological IDH2 inhibitor AGI-6780 displayed synergistic cytotoxicity in MCL and BL cell lines in combination with the proteasome inhibitor carfilzomib, mediated by the blockade of tricarboxylic acid cycle and the decrease in ATP levels, as a consequence of enhanced IDH2 enzymatic inhibition (Bergaggio et al., 2019). Thus, although activating mutations of IDH genes are rare in B-NHL, there may be some room to evaluate, alone or in combination with standard chemotherapy, some of the molecules exhibiting clinical activity in non-lymphoid patients.

## Combination Involving Epigenetic-Targeting Approaches

### Concomitant Targeting of Different Epigenetic Modulators

**Figure 4:**
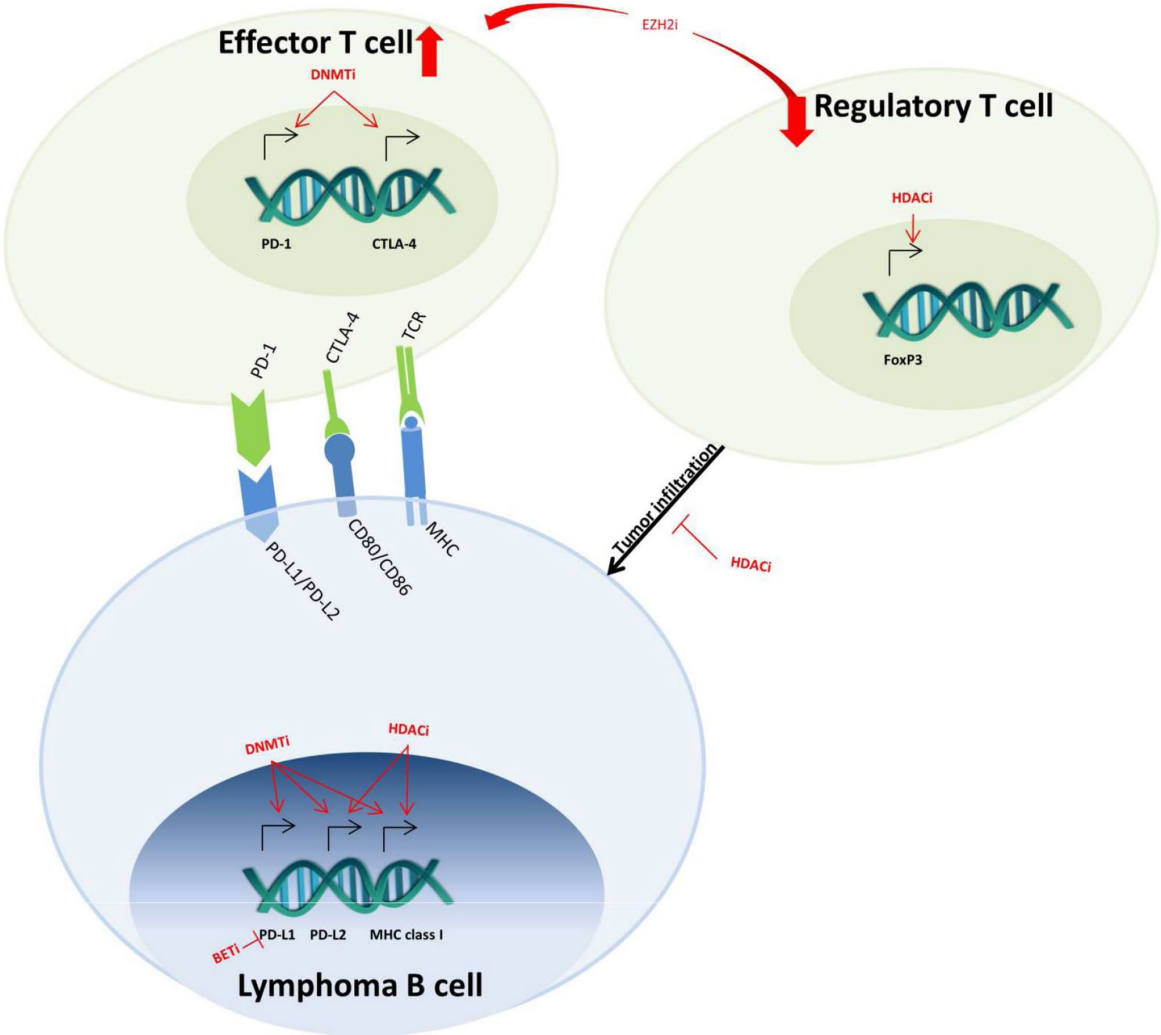
Epigenetic-targeted effects on immuno-oncology mechanisms. In lymphoma B cells, DNMT, HDAC, and BET inhibitors (DNMTi, HDACi, BETi) regulate the expression of MHC class I and PD-1 ligands (PD-L1 and PD-L2). In effector T cells, DNMTi also upregulates the expression of PD-1 and CTLA-4, which leads to T-cell exhaustion. The effects of HDACi on FoxP3 decrease the infiltration of regulatory T cells into the tumor. Lastly, EZH2i decreases the regulatory and increases effector T-cell population in the microenvironment.

In recent years, thanks to the many works directed to characterize and get a better understanding of the human epigenome, it came out that more than 50% of the human cancers account for aberrant changes in chromatin organization at certain genomic regions, as a consequence of mutations in enzymes involved in the regulation of chromatin structure (You and Jones, 2012; The Cancer Genome Atlas Research Network, 2013). Changes in the activity of these chromatin modifiers can lead not only to the initiation of a tumor formation process but also to its progression, metastasis, development of drug resistances, and further relapse and/or escape from immune surveillance (Jones et al., 2016). Therapeutic modulation of such alterations can be achieved with chemical compounds that broadly affect the structure of the DNA such as DNMTis, histone HDACis, or BETis (**Figure 4**). While single-agent clinical trials with these compounds have been conducted with some success in MDS or R/R AML patients receiving azacitidine (Scott, 2016; Schuh et al., 2017) or in R/R FL, MZL, and MCL patients treated with vorinostat (Kirschbaum et al., 2011; Ogura et al., 2014), the association of these agents with other compounds has also been tested. As an example, the combinatorial treatment with vorinostat and the sirtuin inhibitor niacinamide was evaluated in R/R NHL and HL cases (NCT00691210) (Amengual et al., 2013), but it achieved a modest efficiency with an ORR below 50% (Olsen et al., 2007). Other examples include the combination of panobinostat with decitabine which displayed synergistic caspase-dependent cell death in DLBCL cells (Kalac et al., 2011) or the combination of romidepsin with the antimetabolite pralatrexate for the treatment of relapsed PTCL (Amengual et al., 2018).

A different therapeutic approach consists in targeting specifically certain chromatin regulatory proteins to achieve a more restricted effect in the transcription of a concrete subset of genes. Promising examples are the inhibition of the DOT1-like (DOT1L) histone H3K79 methyltransferase with pinometostat (EPZ-5676) in adults with MLL/KMT2A-driven leukemia (NCT02141828) (Stein et al., 2018) or inhibition of histone H3K4 and K9 demethylation by the lysine-specific demethylase 1 (LSD1) inhibitor seclidemstat, currently being assessed in clinical trials to treat refractory Ewing sarcomas (NCT03600649).

Combinations with chemical compounds that broadly affect an epigenetic mark and a specific inhibitor of a chromatin-modifying enzyme, such as the EZH2 inhibitor GSK126 and romidepsin, have also been assessed in preclinical studies with DLBCL-GCB cell lines, leading to synergistic tumor growth inhibition effects in mice (Lue et al., 2019). Another example of the strategies currently evaluated in clinical studies is the concomitant treatment of drug-resistant MM with panobinostat and bortezomib (NCT01083602) (Richardson et al., 2016).

Finally, and in concordance with the concept that acquired resistance to chemotherapy is tightly linked to changes in chromatin structure, many efforts have been made in identifying combinational strategies associating different types of cytotoxic drugs to small molecule regulators of chromatin modifiers. As an example, the dinitroazetidine derivative RRx-001 administered in combination with radiation, chemotherapy, or immunotherapies promotes the generation of reactive oxygen and nitrogen species, leading to the oxidation of the cysteines present at the catalytic sites of DNMTs and HDACs. This phenomenon entrains the inhibition in DNMT and HDAC enzymatic activities, with subsequent alterations in the chromatin structure. The therapeutic benefits of this compound have been assessed in phase II clinical trials both as a radio- and chemo-sensitizer, as well as a way to prone tumor response to conventional therapies (NCT02215512, NCT02452970, NCT02096341, NCT02871843) (Oronsky et al., 2017; Zhao et al., 2017).

### Combination of Epigenetic Drugs With Other Classes of Anti-Tumoral Drugs

**Figure 5:**
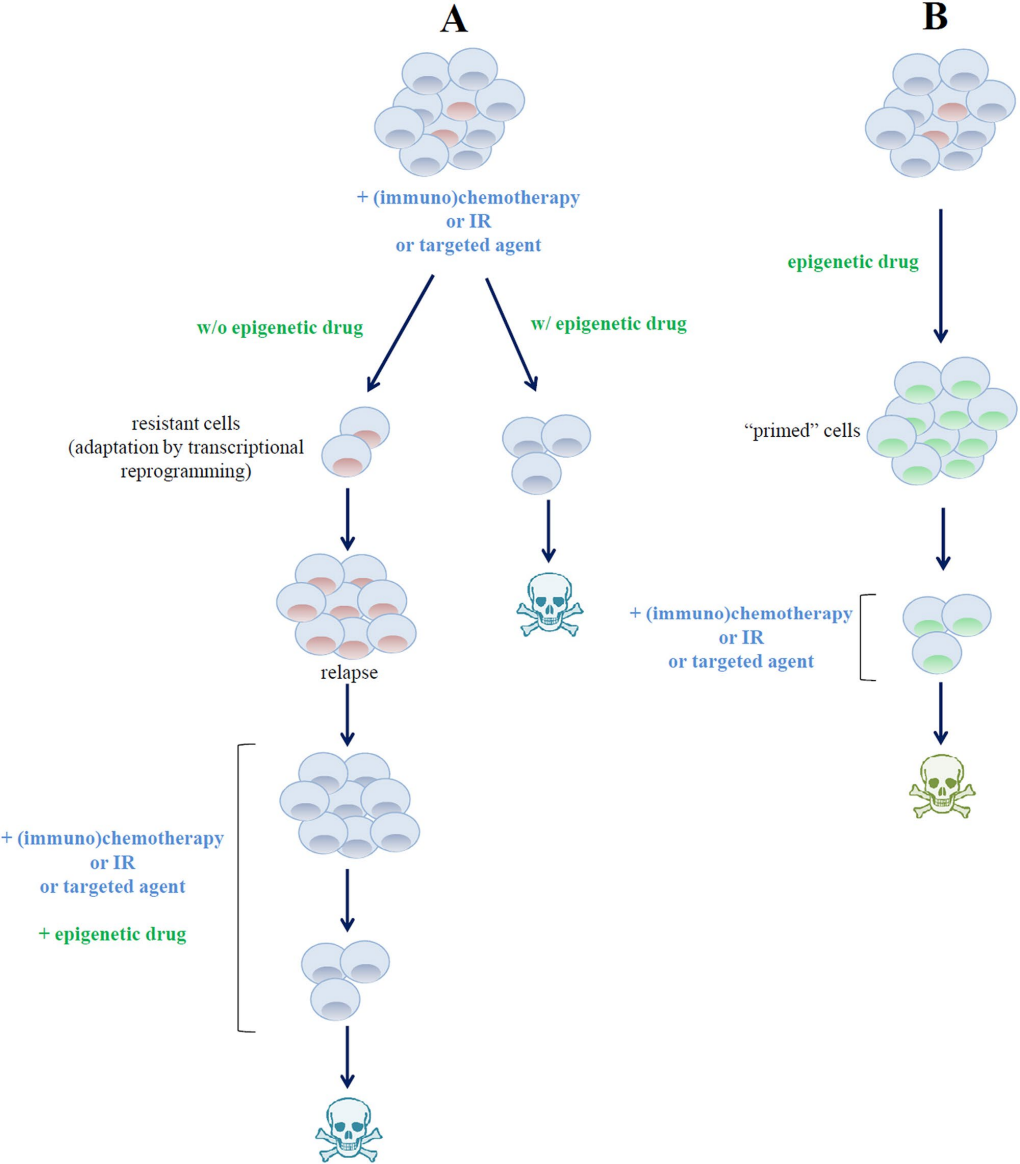
Different strategies for combining drugs targeting epigenetic regulators in B-cell lymphoma. **(A)** Epigenetic drugs can be used to overcome (left axis) or to circumvent (right axis) malignant B-cell resistance to targeted agents or to standard chemotherapeutic regimens. **(B)** Pre-exposure of tumor cells to epigenetic drugs induces profound modifications of cell transcriptional profile, thus priming them to the cytotoxic effect of chemotherapeutic and targeted agents.

The use of epigenetic agents combined with other anti-tumoral drugs may represent the future of epigenetic-targeted therapies (**Figure 5**). The rationale of such combinations would be, on the one hand, to benefit from the transcriptional effects of targeting epigenome. Indeed, growing evidences are showing that epigenetic therapy, using DNMTi or HDACi, in combination with conventional therapy or immunotherapy, might be an up-and-coming step toward the development of new and efficient cancer treatment strategies (Brahmer et al., 2012; Sharma and Allison 2015; Topalian et al., 2015; Issa et al., 2017). Accordingly, the acquired capacity of tumors to resist chemotherapy is related with changes in the cancer cell’s epigenome, which might affect directly the cell cycle and/or some key apoptosis regulators (Fodale et al., 2011).

In a phase I study, Clozel and collaborators proposed a new approach to beaten chemotherapy resistance in DLBCL patients. The authors demonstrated a high rate of complete remission when a 5-day exposure to azacitidine followed by treatment with R-CHOP was employed. Mechanistically, the treatment leads to the demethylation of the chemoresistance-associated gene SMAD1 and subsequent chemosensitization (Clozel et al., 2013). Based on these results, an ongoing phase I study using azacitidine combined with R-CHOP in therapy-naive DLBCL, grade 3B FL, or transformed FL patients is showing promising preliminary results (NCT02343536). Finally, the safety and tolerability of adding oral azacitidine to R-ICE therapy is being evaluated in R/R DLBCL patients (NCT03450343).

Regarding HDACi, *in vitro* studies have demonstrated that this class of agents can synergize with chemotherapy. Globally, these trials have had mixed heterogeneous results. Among the potently successful studies, in indolent B-NHL the vorinostat/rituximab combination exhibited a nice activity with an acceptable safety profile and durable responses (Chen et al., 2015). Ageberg and collaborators also showed that valproic acid sensitizes to CHOP and enhances the CHOP ability to induce apoptosis in DLBCL cell lines (Ageberg et al., 2013). Subsequently, it has been shown in a small set of DLBCL patients that the administration of valproate before R-CHOP treatment upregulated the CD20 levels and increased the efficacy of anti-CD20-based therapy (Damm et al., 2015). Recently, the VALFRID phase I trial (NCT01622439) showed that valproate when added to standard R-CHOP therapy is secure, tolerable, and increases OS in DLBCL patients (Drott et al., 2018). The efficacy of vorinostat combined with cyclophosphamide, etoposide, and prednisone (R-CVEP) was evaluated in aged patients with R/R DLBCL (NCT00667615); however, the R-CVEP association did not reach the criteria for cohort expansion (Straus et al., 2015). Similarly, the combination of vorinostat with R-CHOP was evaluated in the SWOG S0806 phase I/II trial (NCT00972478) without success in DLBCL patients (Persky et al., 2018). Panobinostat was tested in combination with conventional therapy and although the data from the clinical trial NCT01238692 suggested that as a single agent this drug induces a durable response in a subset of R/R DLBCL patients, its combination with rituximab did not improve the response rate (Assouline et al., 2016). Similarly, Barnes and collaborators observed that this combination was effective in a minority of DLBCL patients heavily pretreated (NTC01282476) (Barnes et al., 2018). The combination with immunomodulatory drug (IMiD) lenalidomide was assessed in a phase I/II clinical trial in patients with R/R HL (NCT01460940); however, the combination was not advantageous over single-agent treatment and raised relevant concerns regarding the toxicity (Maly et al., 2017). Finally, preclinical data have shown that belinostat exhibits synergistic cytotoxic activity in DLBCL cell lines when associated to the microtubule-interfering drug vincristine, mediated by the prevention of cell polyploidy (Havas et al., 2016).

Regarding EZH2 inhibitors, combinatorial treatments with tazemetostat and the anti-programmed death-ligand 1 (PDL1) antibody atezolizumab (NCT02220842), prednisone alone, or combined with other components of CHOP regimen are currently being evaluated in patients with refractory DLBCL (NCT02889523) (Gulati et al., 2018). Moreover, combinations with EZH2 inhibitors and inhibitors of the BCR signaling cascade such as ibrutinib, the spleen tyrosine kinase (SYK) inhibitor tamatinib, the mammalian target of rapamycin (mTOR) inhibitor everolimus, or MAPK inhibitor have also been challenged in pre-clinical models of DLBCL (Brach et al., 2017; Lue et al., 2017). Other therapeutic strategies currently assessed in pre-clinical studies for the treatment of MM consist in combining the inhibitor tazemetostat with IMiDs such as lenalidomide or pomalidomide (Dang et al., 2016), glucocorticoid receptor agonists (dexamethasone or prednisolone), proteasome inhibitors (bortezomib or ixazomib) (Drew et al., 2017), or HDACis (Issa et al., 2017).

Finally, in combination with the CDK4/6 inhibitor palbocilib, the BETi JQ1 has shown synergistic activity in MCL *in vitro* and *in vivo* (Sun et al., 2015). Another member of the CDK family, CDK9, is a core component of the assembly of the positive transcription elongation factor complex (P-TEFb), which is recruiting by BRD4. In relation with this, the BETi BI-894999 shows profound synergy with CDK9 inhibitors alvocidib and LDC000067 in both *in vitro* and *in vivo* models of hematological malignancies (Doroshow et al., 2017). Among other promising combinations, CPI203 combined with the proteasome inhibitor bortezomib or with lenalidomide was particularly efficient in aggressive bortezomib-resistant MCL tumors (Moros et al., 2014), and GS-5829 synergistically interacted with venetoclax or with BCR-interfering agents in preclinical models of DLBCL, MCL, and/or CLL (Bates et al., 2016; Kim et al., 2017).

## Conclusions

Besides the well-known genomic changes, several epigenetic modifications that result in an altered chromatin state and alterations in the DNA methylation status have been described in lymphoma cells. In general, these alterations favor the malignant transformation and/or tumor progression. Among the mechanisms that may apply to several lymphoma entities, epigenetic activation of suppressors of lineage fidelity leads to downregulation of lineage-specific genes, while additional silencing of essential transcription factors through H3K27 trimethylation avoids the restoration of the cell type characteristic expression program. Therefore, there is undoubtedly an important clinical role for epigenetic drugs across the spectrum of lymphoid malignancies, including B-NHL.

In the last decade, the progresses in the awareness of epigenetic changes in lymphoma cells have paved the way for targeted therapy alternatives employing epigenetic drugs. Treatment approaches such as HDAC inhibition or DNMT blockade have shown remarkable activity in specific subsets of lymphoma patients who remained unresponsive to or relapsed after standard therapy. These drugs have already been added into routine use for patients with a particular lymphoma/leukemia subtype and are the most broadly studied now. However, the identification of biomarkers of clinical sensitivity/resistance to these agents is still needed in order to better identify those lymphoma patients suitable for treatment with these drugs, and for the design of rationally based targeted combination therapies. Although several epigenetic drugs can be successfully combined with standard chemotherapy, allowing to decrease the chemotherapy doses and to limit toxicities and adverse effects, co-administration of two epigenetic modulators like DNA hypomethylating agents and HDAC inhibitors, for example, can also show synergistic molecular effects, resulting in increased antitumor activity.

In the light of the large number of drugs currently in clinical development in B-NHL patients, selection of the most relevant targeted therapies will be extremely important to move the field ahead. Epigenetic drugs with more specific targets, such as EZH2 inhibitors or BRD4 inhibitors, but also the newer epigenetic agents like PRMT5 and IDH inhibitors, are also of great interest, as demonstrated by a particularly rapid translation from bench to bedside within the past 5 years.

Despite these considerable advances in epigenetic drug therapy in B-cell lymphoma, there is still some way to go before reaching a complete overview of the complex landscape of the epigenetic modifications occurring during the lymphomagenesis, and much work is still to be done to improve the rationale use of epigenetic drugs in lymphoma patients. According to the promising reports from several trials involving the newest agents and the most innovative drug combinations in B-NHL patients with relapse disease, it seems that we are entering a very exciting era for the field of epigenetics in lymphoma.

## Author Contributions

MR, DR, MA, MF and GR made a substantial contribution to all aspects of the preparation of this manuscript.

## Funding

The authors received financial support from Fondo de Investigación Sanitaria PI15/00102 and PI18/01383, European Regional Development Fund (ERDF) “Una manera de hacer Europa” (to GR). The authors received fundings from TG Therapeutics and Celgene Corp to support researches unrelated to the present work. Funders were involved neither in the design, nor in the writing of this review.

## Conflict of Interest

The authors declare that the research was conducted in the absence of any commercial or financial relationships that could be construed as a potential conflict of interest.
